# Detection of Podocin in Human Urine Sediment Samples by Charge Derivatization and LC-MS-MRM Method

**DOI:** 10.3390/ijms21093225

**Published:** 2020-05-02

**Authors:** Remigiusz Bąchor, Dorota Gąszczyk, Karolina Panek-Laszczyńska, Andrzej Konieczny, Wojciech Witkiewicz, Piotr Stefanowicz, Zbigniew Szewczuk

**Affiliations:** 1 Faculty of Chemistry, University of Wroclaw, 50-383 Wroclaw, Poland; dorota.gaszczyk@chem.uni.wroc.pl (D.G.); piotr.stefanowicz@chem.uni.wroc.pl (P.S.); zbigniew.szewczuk@chem.uni.wroc.pl (Z.S.); 2 1st Department and Clinic of Gynecology and Obstetrics, Wroclaw Medical University, 50-368 Wroclaw, Poland; karolina.panek@gmail.com; 3 Department of Nephrology and Transplantation Medicine, Wroclaw Medical University, 50-556 Wroclaw, Poland; andrzej_konieczny@yahoo.com; 4 Research and Development Center, Regional Specialized Hospital, 51-124 Wroclaw, Poland; witkiewicz@wssk.wroc.pl

**Keywords:** podocin, podocyturia, preeclampsia, charge derivatization, LC-MS, MRM

## Abstract

Detection of podocytes in urine might serve as a useful diagnostic tool in both primary and secondary glomerular diseases. The utility of podocyturia has been confirmed for both pre-eclampsia and glomerulonephritis. Here, we present a new and sensitive method for qualitative LC-MS-multiple-reaction-monitoring (MRM) analysis of podocin, serving as a podocyturia biomarker in urine sediments. The following podocin tryptic peptides with the ^169^LQTLEIPFHEIVTK^182^, ^213^AVQFLVQTTMK^223^, ^240^SIAQDAK^246^, and ^292^MIAAEAEK^299^ sequences were applied as a model. The selective chemical derivatization of the ε amino group of C-terminal lysine residue in tryptic peptides, by 2,4,6-triphenylpyrylium salt (TPP) as a fixed charge tag, was employed to increase the ionization efficiency, in routine ESI-MS analysis. Additionally, the generation of a reporter ion, in the form of a protonated 2,4,6-triphenylpyridinium cation, makes the derivatized peptide analysis in the MRM mode unambiguous. Identification of derivatized and non-derivatized peptides were performed, and the obtained results suggest that the peptide with the ^292^MIAAEAEK^299^ sequence may serve as a marker of podocyturia.

## 1. Introduction 

Podocyturia refers to the presence of podocytes, highly differentiated glomerular epithelial cells, maintaining the filtration barrier located in the outer space of the glomerular basement membrane (GBM) in urinary sediment [1]. Podocytes detected in urine are viable despite the lack of hostile glomerular environment and urinary acidity. Podocyturia may precede proteinuria, the traditional marker of glomerular injury, and reflect the disease activity. Therefore, in diseases affecting glomeruli, the presence and the quantification of urinary podocytes may be of remarkable relevance as it can herald the appearance of proteinuria. 

Preeclampsia (PE) is a severe pregnancy-related disorder affecting a large number of pregnant women, with delivery as the only cure [2,3,4]. Ten million women around the world develop PE each year and 76,000 of them die each year. The number of newborns dying because of this disorder is estimated at 500,000 per annum [5]. Symptoms of PE are defined as a presence of systolic blood pressure ≥ 140 mmHg and/or diastolic blood pressure ≥ 90 mmHg, as measured twice at least 6 h and less than 7 days apart, with the coexistence with one or more of the following new-onset conditions: Proteinuria, renal insufficiency, liver involvement, neurological complications, hematological complications, or uteroplacental dysfunction (fetal growth restriction) [6,7]. Although the absence of proteinuria does not exclude the appearance of PE, glomerular injury still plays a crucial role in its pathogenesis. The presence of podocytes, as well as podocyte-specific proteins (such as nephrin, synaptopodin, and podocin), in urine of pregnant women, suggests a possible appearance of PE at an early stage [8,9]. Currently, podocyte detection is a potential and promising candidate for being applied as a marker for diagnosis, treatment, and prevention of PE’s long-term complications [10]. It was also demonstrated that both nephrin and synaptopodin expression were reduced in contrast to the intact expression of podocin in women affected by PE [11]. Podocin has been determined as the most robust podocyte marker, compared to nephrin and synaptopodin, with 100% sensitivity in women with PE. Therefore, podocin is considered as one of the most podocyte-specific biomarkers of PE. 

Previously, we published a review describing identification methods of podocyte injury biomarkers in urine [12]. They involve a cell culture, accompanied with immunofluorescence, a quantitative polymerase chain reaction, and other immunoanalytical-based methodologies such as ELISA. However, all of the presented methods have some significant drawbacks such as the generation of antibodies [9]. In 2013, Garovic et al. [13] presented the application of mass spectrometry as a new diagnostic tool of podocyturia detection, in urine samples, among women with diagnosed PE. In this method, the pellet was digested by trypsin and the obtained hydrolysates were analyzed by the LC-MS-multiple-reaction-monitoring (MRM) technique, in the presence of isotopically labeled internal standard. The podocin tryptic fragment, with the ^39^QEAGPEPSGSGR^50^ sequence, was described as the most promising for podocin quantification. Additionally, achieved results were confirmed by ELISA tests. The analysis was performed using 50 mL of urine sample. Another approach was presented by Simon et al. [14]. In their study, podocin was quantified in the soluble fraction of urine. The tryptic podocin peptide, with the ^59^APAATVVDVDEVR^71^ sequence, was chosen for podocin quantification by LC-MS-MRM. The described results clearly confirmed the usefulness of the LC-MS-MRM method as a diagnostic tool for podocin detection in urine. The ESI-MS analysis of tryptic peptides, containing lysine residue at the C-terminus, may be difficult for MS analysis due to their low ionization efficiency. Therefore, increasing the sensitivity of this method may allow analysis of trace amounts of the biomarkers in the early stage of preeclampsia. Additionally, Martineau and co-workers [15] used the ultra-performance liquid chromatography tandem mass spectrometry technique for simultaneous quantitation of podocalyxin and podocin in urine specimens.

We also applied the LC-MS-MRM technique, in analysis of podocin, in tryptic digests of feline [16] and canine [17] urine samples. The evaluated group of animals consisted of cats and dogs with diagnosed chronic kidney disease. The performed study has proved that the proposed method may be applied in the analysis of podocin in animal urine samples. Based on previous results, further research was conducted, using horse urine samples, because there are no biomarkers in this species specific for diagnosing acute kidney injury (AKI) in the asymptomatic stage. However, to increase the sensitivity, the proposed method needs further optimization.

Recently, we developed a method of increasing the sensitivity of tryptic peptides by mass spectrometry analysis, by their derivatization with ionization enhancers, containing the quaternary ammonium group and their analysis by tandem mass electrospray mass spectrometry, hyphenated with liquid chromatography (LC-ESI-MS/MS) [18]. The fixed charge tag proposed by us, in the form of 2,4,6-triphenylpyrylium salt, in the reaction with the ε-amino group of lysine residue, resulted in the formation of the 2,4,6-triphenylpyridinium derivative [19,20]. Such a charge tag increases the ionization efficiency during mass spectrometry analysis, making possible the sensitive analysis of trace numbers of compounds. The application of inexpensive and commercially available pyrilium salt as a derivatization reagent allows selective derivatization of the ε-amino group of lysine, containing peptides present in complex mixtures. Peptides modified by the 2,4,6-triphenylpyridinium group in MS/MS experiments generate the protonated 2,4,6-triphenylpyridinium ion, serving as a reporter ion in multiple-reaction-monitoring (MRM) mode. Moreover, the developed derivatization of peptides is inexpensive and easy to perform, even by an inexperienced analyst. The 2,4,6-triphenylpyrylium salt was also applied for sensitive ESI-MS detection of cysteine-containing peptide, captured by properly designed solid support [21].

In this manuscript, we propose a new and sensitive method of tryptic podocin peptide identification in urine sediments, based on the sample derivatization by a fixed charge tag, in the form of 2,4,6-triphenylpyrylium salt and the targeting LC-MS-MRM technique. 

## 2. Results 

**Figure 1:**
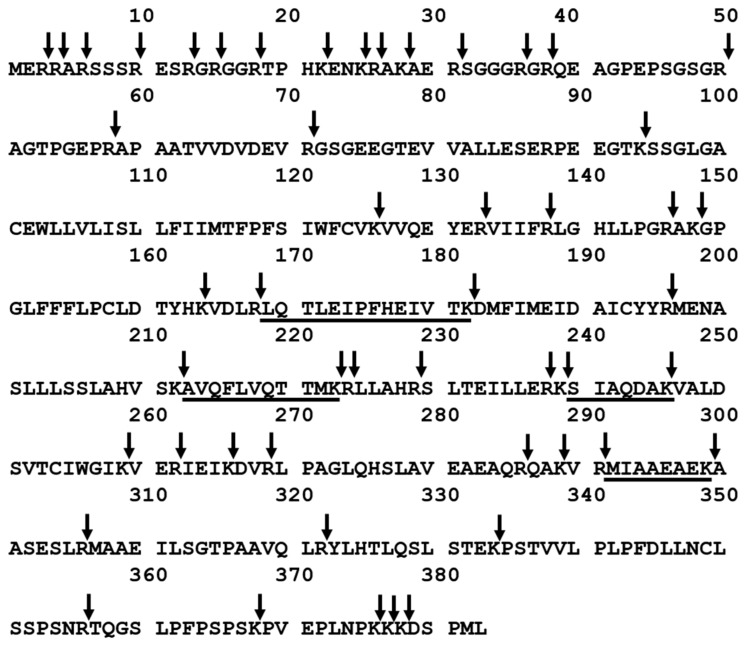
Sequence of human podocin with marked trypsin cleavage sites determined according to the UniProt databases (entry name PODO_HUMAN). The selected sequences were underlined. Arrow indicates the trypsinolysis site.

The aim of this work was to investigate the applicability of podocin tryptic peptide charge derivatization, by 2,4,6-triphenylpyridinium salt, to increase their ionization efficiency, allowing ultrasensitive detection by the LC-MS-MRM method. The tryptic human podocin peptides with the ^169^LQTLEIPFHEIVTK^182^, ^213^AVQFLVQTTMK^223^, ^240^SIAQDAK^246^, and ^292^MIAAEAEK^299^ sequences were chosen as potential podocin biomarkers (Figure 1). According to the Protein Information Resource Database (https://proteininformationresource.org/pirwww/index.shtml), all of the peptides are present only in human podocin. 

**Figure 2:**
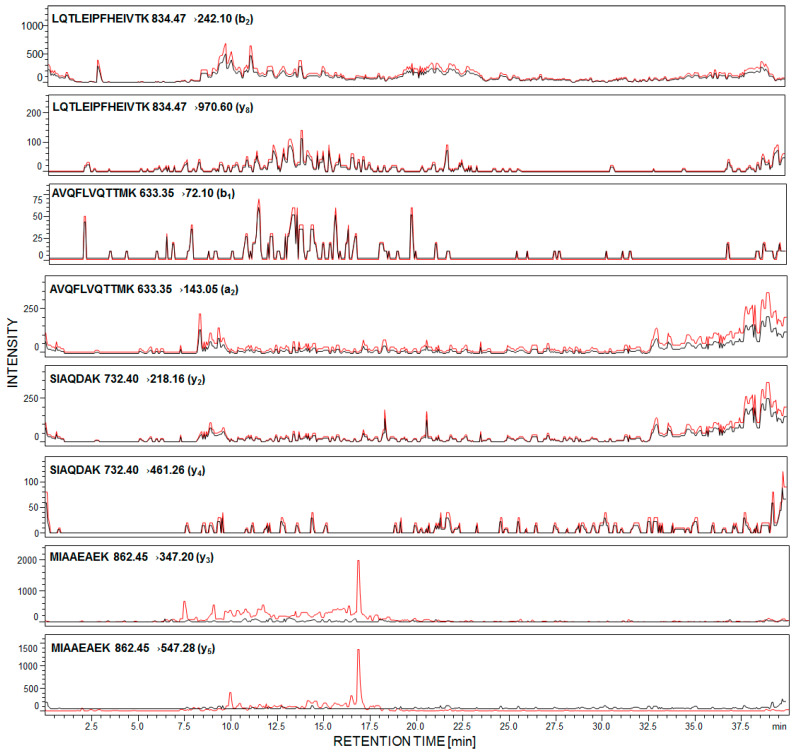
Multiple-reaction-monitoring (MRM) analysis of urine sediment tryptic digest from healthy subjects (black line) and pregnant women with diagnosed preeclampsia (red line).

First, we determined the detection sensitivity of model synthetic peptides, using LC-MS/MS methods. The data revealed that the ^169^H-LQTLEIPFHEIVTK-OH^182^ peptide may be analyzed even at the subfemtomolar level of detection (6.0 × 10^−16^ mole). Other peptides were identified at 1.2 × 10^−15^ mole for ^292^MIAAEAEK^299^, 6.4 × 10^−15^ mole for ^240^SIAQDAK^246^, and 7.9 × 10^−15^ mole for ^213^AVQFLVQTTMK^223^. The performed experiment allowed peptides to be chosen with the ^169^H-LQTLEIPFHEIVTK-OH^182^ sequence as a potential model for sensitive detection and identification of podocin in human urine sediment samples. For each of the synthesized peptides, the MRM methods were optimized and the following transitions were applied, for podocin investigation: For ^169^LQTLEIPFHEIVTK^182^ [M + 2H]^2+^ (*m*/*z* 834.47) ion, as the most intensive signal on the obtained mass spectra, 834.47→242.10 (b_2_), 834.47→970.60 (y_8_); for ^213^AVQFLVQTTMK^223^ [M + 2H]^2+^ (*m/z* 633.35) ion, as the most intensive signal on the obtained mass spectra, 633.35→72.10 (b_1_), 633.35→143.05 (a_2_); for ^240^SIAQDAK^246^ 732.40→218.16 (y_2_), 732.40→461.26 (y_4_); for ^292^MIAAEAEK^299^ 862.45→347.20 (y_3_), 862.45→547.28 (y_5_). The urine sediment samples were prepared according to the procedure described in the Section 4. The achieved results for the sample of healthy subjects (black line) and with diagnosed PE (red line) are presented below (Figure 2). 

In the case of healthy individuals, signals corresponding to the investigated peptides are absent (Figure 2, black line). It was found that urinary podocyte excretion may occur in the case of healthy patients (<0.5 podocytes/mg creatinine) [22]; however, the number of released cells and podocin may not be sufficient for the identification by commonly used analytical techniques. Garovic and co-workers claimed [23] that podocytes were absent in urine among healthy pregnant women and those with gestational hypertension but without other symptoms of PE.

The presented MRM chromatograms for all of the selected and synthesized tryptic human podocin peptides show only signals corresponding to MRM transitions of peptide, with the ^292^MIAAEAEK^299^ sequence, in the urine sediment sample from pregnant woman with diagnosed PE (Figure 2, red line). Signals corresponding to the fragment ion of other analyzed peptides have not been identified. There are two possible explanations of such an observation. First, the tryptic digestion of human podocin may result in the formation of a small number of these peptides or have not even been formed at all. Another possibility is that podocin may exist in two isoforms – canonical and an ill-defined short one. A shorter 315 amino acid isoform of human podocin lacks one exon encoding the central part of the prohibition domain (PHB-domain) [24,25]. The presence of a short podocin isoform was determined by Volker and co-workers using mass spectrometry [26]. The role of the short isoform of podocin is still unknown and may differ from the canonical variant. It was suggested that the short isoform may affect the lipid and protein composition of the slit diaphragm. Based on the sequence of the short human podocin isoform (UniProt identifier: Q9NP85-2), in the missing fragment, with the ^180^VTKDMFIMEIDAICYYRMENASLLLSSLAHVSKAVQFLVQTTMKRLLAHRSLTEILLERKSIAQDAKV^247^ sequence, the chosen model peptides with the ^169^LQTLEIPFHEIVTK^182^, ^213^AVQFLVQTTMK^223^ and ^240^SIAQDAK^246^ sequences were included. Additionally, according to the Protein Information Resource Database, sequences presented above are characteristic only for the canonical podocin isoform, whereas the ^292^MIAAEAEK^299^ sequence is characteristic for both the canonical and short podocin isoform. Therefore, whether the short isoform of podocin is present in PE, the identification of the presented model peptides is not possible. However, there are no literature data describing which podocin isoform is more characteristic for podocyturia. Additionally, both Garovic and co-workers [11] and Simon et al. [14] described the tryptic human podocin peptide sequences, which may be used as a podocin marker, based on the sequence and trypsynolysis of the short podocin isoform. Therefore, in our work, the applicability of other tryptic podocin fragments, originating from the canonical form, as a potential podocin marker, which might be identified by LC-MS-MRM, was the main goal. 

**Figure 3:**
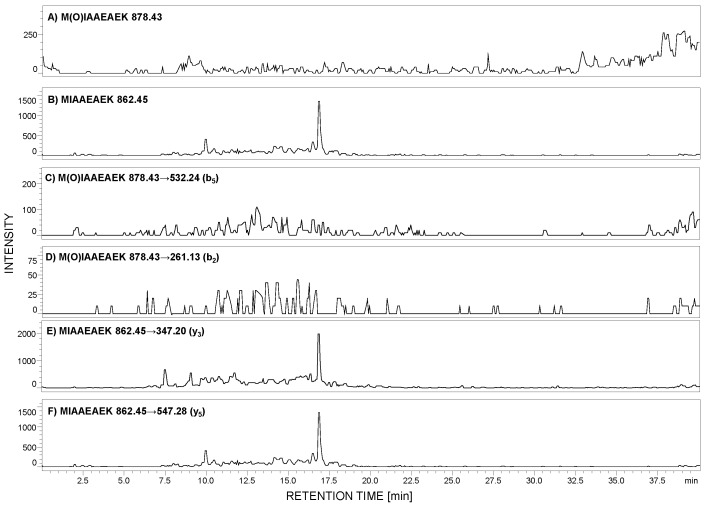
Selected ion monitoring of M(O)IAAEAEK (**A**) and MIAAEAEK (**B**) peptide sequences in urine sample tryptic digest from patient with diagnosed preeclampsia (PE). MRM investigation of M(O)IAAEAEK (**C**,**D**) and MIAAEAEK (**E**,**F**) peptide sequences in urine sediment tryptic digest from pregnant women with diagnosed preeclampsia. MRM transitions corresponding to the oxidized peptide with the M(O)IAAEAEK sequence were not identified.

According to the peptide selection criteria for proteomic analysis, presented by Mohammed and co-workers [27], methionine residue should be absent in the peptide of interest due to the possible oxidation to sulfoxide (+16 Da mass shift), which may reduce the sensitivity during MRM analysis. To check the stability of the selected peptide, with the ^292^MIAAEAEK^299^ sequence, under the conditions applied during sample preparation, the 0.1 mg of this peptide was added to the urine sediment sample. After all steps, the LC-MS analysis was performed in the selected ion monitoring mode to analyze the possibility of methionine oxidation. The *m*/*z* values for the native (862.45 [M + H]^+^) and oxidized form (878.43 [M + H]^+^) were monitored (Figure 3A,B). The obtained results clearly confirmed that under the applied conditions, the model peptide did not undergo oxidation.

Additionally, the obtained sample was stored at room temperature for one week, and even after this time, methionine oxidation was not observed. To clearly demonstrate the lack of methionine oxidation, under the applied conditions during the sample preparation, we obtained the sulfoxide derivative of the model peptide, developed the MRM method, and the following transitions 878.43→532.24 (b_5_) and 878.43→261.13 (b_2_) were monitored in the urine sample, in which the native form of the MIAAEAEK peptide was identified (Figure 3C–F). The achieved results clearly confirmed the lack of oxidation. Therefore, it may be concluded that the proposed peptide may be used in the podocin investigation in urine sediment samples.

**Figure 4:**
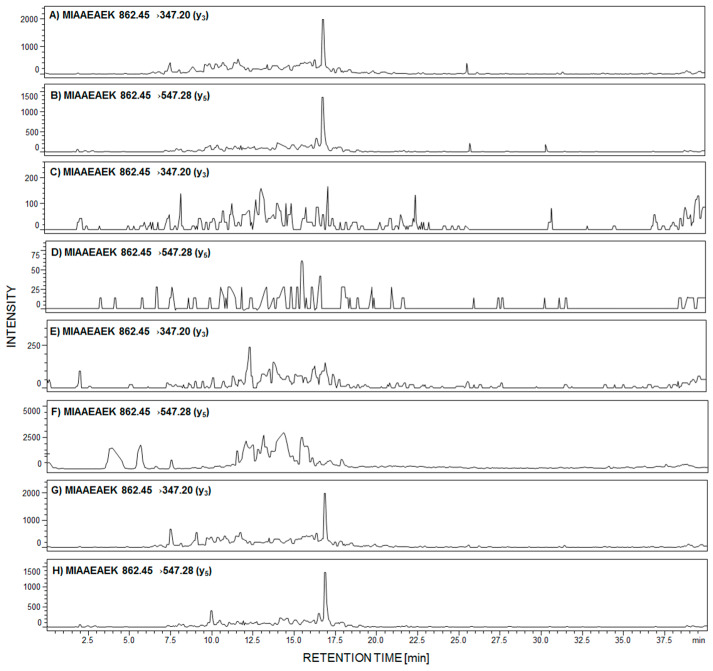
MRM investigation of ^292^MIAAEAEK^299^ peptide in urine sediment tryptic digest from patient with diagnosed membranous nephropathy (MN) (**A**,**B**); focal segmental glomerulosclerosis (FSGS) (**C**,**D**); membranoproliferative glomerulonephritis (MPGN) (**E**,**F**); and IgA nephropathy (**G**,**H**).

Due to the fact that in the performed analysis, only the peptide with the ^292^MIAAEAEK^299^ sequence was identified in the urine sediment sample, from women with PE, we decided to analyze the presence of this tryptic podocin fragment by LC-MS-MRM in the urine sediment samples from patients with diagnosed membranous nephropathy (MN), focal segmental glomerulosclerosis (FSGS), membranoproliferative glomerulonephritis (MPGN), and IgA nephropathy (IgAN). The obtained results are presented in Figure 4A–H. 

The signals corresponding to the MRM transition of fragment ions of the ^292^MIAAEAEK^299^ peptide are present in panels A,B and G,H, obtained after tryptic digest analysis of urine sediment samples from patients with diagnosed MN (Figure 4A,B) and IgAN (Figure 4G,H). On other MRM chromatograms, transitions corresponding to the analyzed peptide were not identified (Figure 4C–F). 

**Figure 5:**
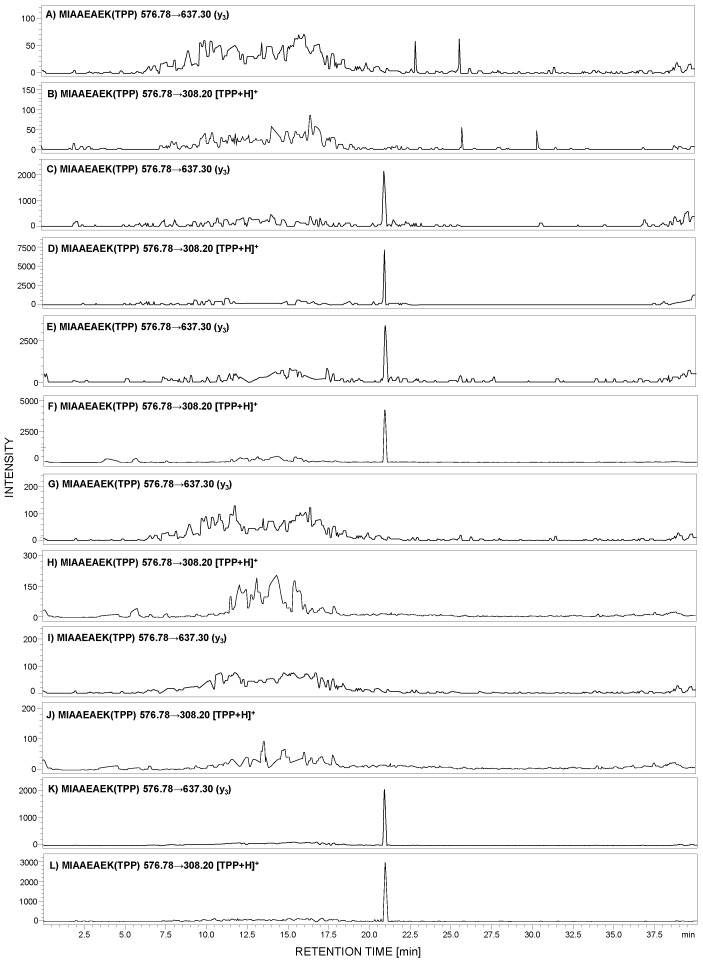
MRM analysis of MIAAEAEK(2,4,6-triphenylpyrylium salt (TPP)) peptide conjugate in tryptic digest of urine sediment samples obtained from healthy patient (**A**,**B**), patient with PE (**C**,**D**), MN (**E**,**F**), FSGS (**G**,**H**), MPGN (**I**,**J**), and IgA nephropathy (IgAN) (**K**,**L**).

Analysis of a small number of peptides by ESI-MS may be limited due to the low ionization efficiency of analytes. Therefore, to overcome this problem, ionization enhancers are commonly used. We decided to apply a strategy, developed by us, of peptide modification by 2,4,6-triphenylpyrilium salt as an ionization marker [15,28]. The model peptide with the ^292^MIAAEAEK^299^ sequence was modified according to the procedure described in the Section 4. The obtained H-MIAAEAEK(TPP^+^)-OH derivative was used for MRM method development. For the selected 2,4,6-triphenylpyrylium salt (TPP)-peptide derivative, the MRM method was optimized using the [M + 2H]^3+^ (*m*/*z* 653.1) ion as the most intensive signal on the obtained mass spectra. The following MRM transitions 576.78→637.30 (y_3_) and 576.78→308.20 ([TPP + H]^+^) from the [M + H]^2+^ ion were selected and used in the analysis of the presence of human podocin in the urine sediment samples. The achieved results for the analyzed samples originating from healthy subjects (Figure 5A,B), a patient with diagnosed PE (Figure 5C,D), membranous nephropathy (Figure 5E,F), FSGS (Figure 5G,H), MPGN (Figure 5I,J), and IgAN (Figure 5K,L) are presented below.

The obtained MRM data (Figure 5) for the urine sediment samples, derivatized by 2,4,6-triphenylpyrilium salt as an ionization marker, present peaks corresponding to the transition characteristic for the selected human tryptic podocin peptide with the ^292^MIAAEAEK^299^ sequence, in the case of the urine sediment sample from pregnant women with PE (Figure 5C,D), MN (Figure 5E,F), and IgAN (Figure 5K,L). The intensive MRM transitions were observed only in these cases, additionally confirmed by the retention time of the ^292^MIAAEAEK(TPP)^299^ conjugate. Moreover, the urine sediment sample from healthy individuals (Figure 5A,B) showed a lack of analyzed tryptic podocin sequence. The same result was observed in the case of patients with diagnosed FSGS (Figure 5G,H) and MPGN (Figure 5I,J). 

The advantage of the applied pyrylium salt, as reagents for ionization, tagging of peptides lies in the high regioselectivity of this reagent – it reacts with sterically unhindered primary amino groups such as the ε-amino group of lysine residue, the fragmentation patterns of labeled peptides, and the possibility of the convenient and economic incorporation of the isotopic label. The 2,4,6-triphenylpyridinium-modified peptides generate an abundant protonated 2,4,6-triphenylpyridinium ion (*m*/*z* 308) in MS/MS experiments. This fragment serves as a reporter ion for the multiple-reaction-monitoring analysis. The intensive signal corresponding to the [TPP + H]^+^ ion was observed by us and is presented in Figure 5. In addition, the fixed positive charge of the pyridinium group enhances the ionization efficiency. Other advantages of the proposed ionization enhancers are the derivatization simplicity of peptides and the possibility of convenient incorporation of isotopic labels into derivatized peptides, which we plan to present in our future manuscript.

In our study, we analyzed the presence of podocin tryptic peptides, with the ^169^LQTLEIPFHEIVTK^182^, ^213^AVQFLVQTTMK^223^, ^240^SIAQDAK^246^, and ^292^MIAAEAEK^299^ sequences, in urine sediment samples from healthy subjects and those with diagnosed preeclampsia, membranous nephropathy, focal segmental glomerulosclerosis, membranoproliferative glomerulonephritis, and IgA nephropathy. We analyzed more than ten samples from healthy pregnant women and other healthy subjects. In the case of subjects with renal dysfunctions, we analyzed five samples for each case. The data obtained after LC-MS-MRM analysis of urine sediment tryptic digests of subjects with diagnosed preeclampsia, membranous nephropathy, focal segmental glomerulosclerosis, membranoproliferative glomerulonephritis, and IgA nephropathy were always the same.

## 3. Discussion

All analyzed samples concerned diseases associated with glomerular injury. Garovic and co-workers reported that the podocyte detection, in the urine of preeclamptic women, may serve as a diagnostic tool in PE due to its strong predictive value [19]. Additionally, authors found that podocin is absent in urine sediment derived from healthy pregnant women and those with gestational hypertension. Furthermore, Aita et al. reported higher urinary podocyte excretion in the third trimester of pregnancy in patients with PE [29]. Therefore, it may be concluded that the received results correlate with the observations presented in the literature. 

MN, a major cause of nephrotic syndrome in adults, is related to the accumulation of immune deposits on the outer aspect of the glomerular capillary wall. In renal biopsies of patients affected by MN, podocyte cell injury and rearrangements of the glomerular basement membrane, leading to proteinuria, were found [30]. The podocytes’ damage causes their detachment and, as a consequence, lead to their presence in urine, enabling their identification. In the case of the analyzed sample (Figure 5E,F), the presence of a signal corresponding to the podocin tryptic peptide confirms the presence of podocin. 

IgAN (Berger’s disease) is most often the primary glomerular disease, related to immunoglobulin A deposition in the glomerular mesangium. Recent literature data suggest a connection between the increased severity of glomerular dysfunction and lower number of glomerular podocytes [31]. The determination of urine podocyte number may serve as a diagnostic tool to differentiate between glomerular and non-glomerular disease and between inflammatory and noninflammatory disease [32]. Our results confirmed such an observation (Figure 5K,L). 

FSGS is another type of primary glomerulonephritis, presenting frequently in form of the nephrotic syndrome. Reidy and Kaskel proved the crucial role of podocyte loss in the FSGS [33]. It was found that podocytes’ damage initiates their detachment from the glomerular membrane [34,35]. It may be assumed that due to their excretion to urine, podocin and its tryptic fragments may be identified in the urine sediment samples [32]. By contrast, Agrawal and co-workers found the reduction of glomerular podocin expression in FSGS [36], which may result in the low level or even lack of this protein in urine. Based on our results, podocin was not observed in the urine sediment samples from patients with diagnosed FSGS. The possible explanation of this may be the fact that FSGS not only reflects the primary affection of glomeruli but might also be a common end-path for other primary glomerular diseases. Possibly, in this case, FSGS reflected to the inactive disease, where proteinuria was only the sign of residual scarring; therefore, we were not able to detect podocin. 

The same result was observed in the case of urine samples from the patient with diagnosed membranoproliferative glomerulonephritis. The lack of MRM transitions on the obtained chromatograms (Figure 4E,F, Figure 5I,J) confirmed the absence of podocin in the analyzed sample. MPGN is a kidney disorder characterized by mesangial cell proliferation and structural changes in glomerular capillary walls [37]. The reduction in glomerular podocin expression in MPGN was also presented [33]. It may also be concluded that in this case, the lack of podocin tryptic peptide, confirmed by the LC-MS-MRM method, in the analyzed urine sediment sample from patients with MPGN has confirmation in literature data. 

## 4. Materials and Methods

### 4.1. Chemicals

All chemicals were used as supplied. Fmoc amino acid and Fmoc-Lys(Mtt)-Wang resin (0.56 mmol/g) were purchased from Novabiochem (Darmstadt, Germany). *N*-[(Dimethylamino)-1*H*-1,2,3-triazolo-[4,5-*b*]pyridin-1-ylmethylene]-*N*-methylmethanaminium hexafluorophosphate N-oxide (HATU) and trifluoroacetic acid (TFA) were obtained from IrisBiotech. Solvents for peptide synthesis (*N*,*N*-dimethylformamide (DMF), dichloromethane (DCM), *N*-ethyldiisopropylamine (DIEA), tetraethylammonium bicarbonate (TEAB) and *N*,*N*,*N*-triethylamine, 1,2-dithiotreitol (DTT), H_2_O_2_, tetrafluoroborate 2,4,6-triphenylpyrylium salt, and iodoacetamide) were obtained from Sigma Aldrich (Saint Louis, MO, USA); triisopropylsilane (TIS) was purchased from Fluka (Bucharest, Romania). Amicon^®^ Ultra Centrifugal Filters were purchased from Merck (Darmstadt, Germany), Rapi-Gest™ SF was from Waters (Waters Chromatography Europe BV, Etten-Leur, Netherlands).

### 4.2. Peptide Synthesis

Model peptides were synthesized manually on Fmoc-Lys(Boc)-Wang resin in Intavis AG syringe reactors containing polyethylene filters, using the Fmoc (9-fluorenylmethoxycarbonyl) solid-phase synthesis procedure [38].

### 4.3. In-Solution Peptide Derivatization with 2,4,6-Triphenypyrilium Salt

The purified model peptides (0.2 mg) were dissolved (in a separate Eppendorf tube) in 0.5 mL of DMF containing 0.05 mg of 2,4,6-triphenylpyrilium tetrafluoroborate (TPP), and 19 μL of *N*,*N*,*N*-triethylamine was added. The mixture was incubated at 60 °C for 1 h and the solution was evaporated under a nitrogen stream. The final product was dissolved in 5% MeCN/H_2_O mixture (*v*:*v*), and lyophilized and used for MS/MS analysis and MRM method development.

### 4.4. Purification

The obtained peptides were purified on a HPLC Thermo Separation system with a YMC-Pack RP C18 column (4.6 × 250 mm, 5 μm), UV detection (210 nm). Elution gradients were 0%–40% *B* in *A* (*A* = 0.1% TFA in water; *B* = 0.1% TFA in acetonitrile/H_2_O, 4:1) for 30 min (flow rate 1 mL/min). The collected fractions lyophilized.

### 4.5. Mass Spectrometry

ESI-MS experiments were performed on a micrOTOF-Q mass spectrometer (Bruker Daltonics, Bremen, Germany) with a standard ESI source. The instruments parameters were as follows: Positive-ion mode, calibration with the Tunemix™ mixture (Agilent Technologies, Palo Alto, CA, USA), mass accuracy was better than 5 ppm, scan range: 50–1600 *m*/*z*; drying gas: Nitrogen; flow rate: 4.0 L/min, temperature: 200 °C; potential between the spray needle and the orifice: 4.2 kV analyte (flow rate: 3 μL/min.

### 4.6. CID

MS/MS experiments were performed for singly ([M + H]^+^) and doubly protonated ([M + 2H]^2+^) precursor ions. Collision gas – argon. The collision energy was optimized between 10 and 30 V to obtain the best quality of fragmentation. Bruker Compass DataAnalysis 4.0 software was used for data analysis.

### 4.7. Urine Sample Preparation 

Collected urine samples obtained from patients with diagnosed membranous nephropathy, focal segmental glomerulosclerosis, membranoproliferative glomerulonephritis (MPGN), and IgA nephropathy (∼1 mL each) were centrifuged (10 min at 5000 rpm, 30 °C). The obtained pellet was used for further experiments. A solid fraction was re-suspended in the mixture of = 0.1 M TEAB (1 mL) buffer containing Rapi-Gest™ SF (0.1%) and sonicated for 5 min. The sample was then centrifuged in an Amicon^®^ Ultra Centrifugal Filter (4000 rpm,15 min). The obtained mixture was diluted with 0.1 M TEAB (1 mL) buffer and centrifuged again. Then, the sample was treated by 100 µL of 0.2 M DTT and incubated for 1 h at 30 °C. After that, the mixture was centrifuged (4000 rpm for 15 min) to remove DTT. Then, 100 µL of 0.1 M iodoacetamide was added and incubated at room temperature for 1 h. Then, the sample was washed with 0.1 M TEAB and centrifuged to remove the excess of iodoacetamide. The obtained supernatant was transferred into the Eppendorf tube, the 50 μg of trypsin in 200 μL of 0.1 M TEAB was added, and the sample was incubated at 37 °C overnight. The enzymatic reaction was terminated by addition of 20 μL of formic acid; after lyophilization, the dry solid was dissolved in 5% MeCN/H_2_O and analyzed by LC-MS.

### 4.8. Urine Tryptic Digest Derivatization with 2,4,6-Triphenypyrilium Salt

The lyophilized tryptic urine hydrolates were dissolved in 0.5 mL of DMF solution containing 0.25 mg of 2,4,6-triphenylpyrilium tetrafluoroborate and 95 μL of *N*,*N*,*N*-triethylamine. The mixture was incubated at 60 °C for 1 h and the solution was evaporated under the nitrogen stream. The final product was dissolved in 5% MeCN/H_2_O mixture (*v*:*v*) and lyophilized.

### 4.9. H_2_O_2_ Oxidation of Model Peptide

Hydrogen peroxide stock (5 µL) was spiked into a 195 µL solution containing 0.1 mg/mL MIAAEAEK peptide in a mildly acidic buffer. The samples were incubated at 37 °C for 2 h, quenched with DTT, dissolved with water (500 µL), and lyophilized. 

### 4.10. Liquid Chromatography - Mass Spectrometry (LC-MS) Analysis in Multiple-Reaction-Monitoring (MRM) Mode

LC-MS/MRM analysis was carried out on a LCMS-8050 Shimadzu apparatus, equipped with a UHPLC Nexera X2 system and Aeris Peptide XB-C18 column (50 mm × 2.1 mm), 3.6 μm bead diameter, at 27 °C. Eluent A: 0.1% formic acid in H_2_O, eluent B: 0.1% formic acid in MeCN. The gradient conditions (B%) were from 5% to 80% B within 40 min. Flow rate: 0.1 mL/min, injection volume: 5 μL. The MRM method was optimized automatically and the following transitions were chosen: ^169^LQTLEIPFHEIVTK^182^ [M + 2H]^2+^ (*m*/*z* 834.47) 834.47→242.10 (b_2_), 834.47→970.60 (y_8_); for ^213^AVQFLVQTTMK^223^ [M + 2H]^2+^ (*m*/*z* 633.35) 633.35→72.10 (b_1_), 633.35→143.05 (a_2_); for ^240^SIAQDAK^246^ 732.40→218.16 (y_2_), 732.40→461.26 (y_4_); for ^292^MIAAEAEK^299^ 862.45→347.20 (y_3_), 862.45→547.28 (y_5_); for M(O)IAAEAEK 878.43→532.24 (b_5_), and 878.43→261.13 (b_2_); for MIAAEAEK(TPP) [M + H]^2+^ 576.78→637.30 (y_3_) and 576.78→308.20. 

## 5. Conclusions

In conclusion, we demonstrated a new method of sensitive LC-MS-MRM analysis of podocin in urine sediments, using selective fixed charge tag derivatization of the ε amino group of C-terminal lysine residue in tryptic peptides. A peptide with the ^292^MIAAEAEK^299^ sequence, characteristic for both the canonical and short podocin isoform, was identified in urine samples from patients with PE, MN, and IgA nephropathy, both in native form and also as a TPP conjugate. The performed study revealed that the selected peptide is stable toward oxidation during the sample preparation steps. It may be assumed that the ^292^MIAAEAEK^299^ peptide sequence may serve as a marker of podocyturia, characteristic for different renal dysfunctions. The proposed strategy opens new possibilities for early diagnosis kidney injury. 

Cysteine-containing tryptic peptide enrichment, using commercially available TentaGel R RAM resin, was modified by the linker containing a maleimide reactive group toward thiol, according to the Michael addition mechanism. The captured peptides containing lysine residue at the C-terminus were modified on the ε-amino group by the quaternary ammonium group in the form of 2,4,6-triphenylpyrylium salt giving 2,4,6-triphenylpyridinium derivative. Such a methodology allowed the sensitive detection of cysteine-containing podocin tryptic peptide. The presented strategy may serve as a potential method in the investigations of PE biomarkers.
